# Multi-view manifold regularized compact low-rank representation for cancer samples clustering on multi-omics data

**DOI:** 10.1186/s12859-021-04220-6

**Published:** 2022-01-20

**Authors:** Juan Wang, Cong-Hai Lu, Xiang-Zhen Kong, Ling-Yun Dai, Shasha Yuan, Xiaofeng Zhang

**Affiliations:** 1 School of Computer Science, Qufu Normal University, Rizhao, 276826 China; 2 School of Information and Electrical Engineering, Ludong University, Yantai, 264025 China

**Keywords:** Low-rank subspace clustering, Concept factorization, Manifold regularization, Cancer multi-omics Data

## Abstract

**Background:**

The identification of cancer types is of great significance for early diagnosis and clinical treatment of cancer. Clustering cancer samples is an important means to identify cancer types, which has been paid much attention in the field of bioinformatics. The purpose of cancer clustering is to find expression patterns of different cancer types, so that the samples with similar expression patterns can be gathered into the same type. In order to improve the accuracy and reliability of cancer clustering, many clustering methods begin to focus on the integration analysis of cancer multi-omics data. Obviously, the methods based on multi-omics data have more advantages than those using single omics data. However, the high heterogeneity and noise of cancer multi-omics data pose a great challenge to the multi-omics analysis method.

**Results:**

In this study, in order to extract more complementary information from cancer multi-omics data for cancer clustering, we propose a low-rank subspace clustering method called multi-view manifold regularized compact low-rank representation (MmCLRR). In MmCLRR, each omics data are regarded as a view, and it learns a consistent subspace representation by imposing a consistence constraint on the low-rank affinity matrix of each view to balance the agreement between different views. Moreover, the manifold regularization and concept factorization are introduced into our method. Relying on the concept factorization, the dictionary can be updated in the learning, which greatly improves the subspace learning ability of low-rank representation. We adopt linearized alternating direction method with adaptive penalty to solve the optimization problem of MmCLRR method.

**Conclusions:**

Finally, we apply MmCLRR into the clustering of cancer samples based on multi-omics data, and the clustering results show that our method outperforms the existing multi-view methods.

## Background

Cancer is a series of complex diseases with high heterogeneity. Nowadays, cancer has gradually become one of the most common and fatal diseases worldwide. Medical studies show that gene variation and mutation are the main factors leading to the formation and development of cancer diseases [1, 2]. Moreover, the abnormality and mutation mechanism of gene will lead to the pathological difference of cancer, thus forming different tumor types. As diagnosis of cancer is very important for the determination of cancer therapeutic schedule or regime, the identification of cancer types has attracted much attention in cancer research [3].

Sequencing technology has opened the omics era of life science and is leading and changing the development of the whole field of cancer research [2, 4]. With the development and popularization of sequencing technology, genomics has made great progress. The generation of massive cancer genomics data provides an effective avenue to investigate the pathogenesis of cancer at the genomic scale. As the most commonly used database for analyzing cancer sequencing data, The Cancer Genome Atlas (TCGA) can provide a variety of cancer genomics data, such as gene expression data, DNA methylation data, copy number variation data, gene regulation data and some clinical medical data [5]. These cross omic measurements provide valuable opportunities for systematic and in-depth study of cancer. In the past decade, TCGA data sets have been widely used in the study of individual cancer type and pan-cancer [6, 7]. And these studies based on TCGA data have contributed to the accumulation and discovery of cancer knowledge.

In the field of bioinformatics, machine learning algorithms play an important role in disease diagnosis, pathogenic factors discovery and treatment outcome prediction, etc. [8, 9]. As an exploratory algorithm in machine learning, clustering algorithm is often used to identify cancer types. In caner classification, the purpose of clustering algorithm is to find sample groups with similar expression patterns by analyzing omics data, so as to classify cancer patients or sample tissues. So far, many classical methods have been proposed for the detection of cancer categories. Gao et al. proposed sparse non-negative matrix factorization to identify cancer class based on gene expression profile [10]. In [11], Ye et al. applied independent component analysis (ICA) into tumor clustering. In [12], the penalized matrix decomposition method was proposed to cluster tumor according to meta samples based on gene expression data. In [13], Nguyen et al. used partial least squares for classification of multiple types of cancer. As in references [10–13], most studies use gene expression data to classify cancer types. With the deepening of cancer research, methylation profile is found to be different among tumor types and can be used as a powerful tool for sample identification [14, 15]. In addition, studies shown that copy number abnormality, as an important gene mutation, can lead to the abnormal growth of tissue cells and play an important role on genetic diversity and evolution [16, 17]. Therefore, these data can also be used as feature sources for cancer type recognition. For example, Polovinkin et al. used DNA methylation data to study the oncological diseases diagnosis, and achieved high accuracy in the classification of different types of cancer patients [18]. Virmani distinguished different subtypes of lung cancer based on DNA methylation markers [19].

All of the above studies indicate that a variety of mutation mechanisms contribute to the occurrence and development of cancer [20]. In order to investigate cancer type identification more accurately, it is necessary to analyze the cancer multi-omics data comprehensively. However, the heterogeneity, high noise, high feature dimensionality and small sample volume, and the differences in measurement and data types of different omics data bring a great challenge to the integrated analysis of multi-omics data [21]. To this end, a variety of integration and analysis algorithms have been proposed. These approaches are mainly divided into two categories. One is network-based methods. For example, Ma et al. presented Affinity Network Fusion (ANF) method to cluster patient using gene expression, miRNA expression and DNA methylation data [22]. Wang et al. developed Similarity Network Fusion (SNF) model to integrate microRNA expression, DNA methylation and mRNA expression data for cancer subtypes identifying [23]. The other is based on matrix decomposition methods. For example, Strazar et al. came up with an Integrative Orthogonality-regularized Nonnegative Matrix Factorization (iONMF) to deal with important information from multiple data sources [24]. Liu et al. presented Block-Constraint Robust Principal Component Analysis (BCRPCA) model to integrate and analysis TCGA data [25].

Recently, the low-rank representation method, namely LRR, was proposed to solve the problem of subspace clustering [26]. In LRR, the rank of representation matrix is considered as equivalent to the dimension of the low-dimensional subspace. LRR strengthens the correlation of representation vectors by enforcing low-rank constraint on the representation matrix. Benefiting from its pleasing efficacy in the acquisition of global structure of high-dimensional data, LRR is considered as a vigorous method and has received a great deal of attention. As a result, many improved methods based on LRR are developed, such as Latent Low-Rank Representation (LatLRR) [27], Structure-Constrained LRR (SC-LRR) [28], Non-negative Spare Hyper-Laplacian regularized LRR (NSHLRR) [29], graph regularized LRR under sparse and symmetric constraints (sgLRR) [30], and Laplacian regularized LRR (LLRR) [31]. However, these methods are only suitable to process single type data. When processing multi-view feature data, these methods may ignore the complementary information between views, thus reducing the learning performance of the algorithm. In order to deal with multi-view data, Brbić et al. developed Multi-view Low-Rank Sparse Subspace Clustering (MLRSSC) [32]. In MLRSSC model, a consistent low-rank affinity matrix is constructed from multi-view data to jointly learn subspace representation. The experimental results on both simulated and real datasets show that MLRSSC method has excellent clustering performance. In [32], it is shown that the MLRSSC framework is suitable for multimodal data, which is crucial to the analysis of heterogeneous multi-omics data. However, MLRSSC method does not consider the influence of local structure on manifold structure learning. Moreover, like most of the existing LRR based methods, it directly uses the observation data as the dictionary matrix to describe the subspaces of data. Since omics data of cancer are usually high-dimension and small sample, using observation data as spatial mapping benchmark will lead to insufficient expression of low-dimensional subspace, thus degrading the learning ability of LRR algorithm on data subspaces.

In light to the shortcomings described above, we present Multi-view Manifold Regularized Compact Low-Rank Representation method, which is called MmCLRR for short. Unlike most LRR based approaches, in MmCLRR, the concept factorization [33] idea is introduced to model dictionary matrix. Specifically, we consider the dictionary as a set of concepts, and each concept corresponds to a low-dimensional subspace, that is, the cluster center. According to concept factorization, the dictionary is modeled as a linear combination of original data. The dictionary matrix constructed by concept can enhance the description of the low-dimensional mapping space and help to obtain the structure of subspace accurately. Besides, the manifold regularization is also imposed on the low-rank affinity matrix to defend the local geometrical structure of each view. Similar to MLRSSC, the ultimate goal of MmCLRR is to achieve the consistent low-rank coefficient matrix from multi-view data. In MmCLRR, we jointly obtain the low-rank representation of multi-view by balancing the consistency of different views. At the same time, the balanced constraint on low-rank representation can avoid the noise propagation in the mapping process.

The key contributions of this study are summarized as below.

1. A multi-view based clustering analysis method named MmCLRR is proposed. Against specified fixed dictionary matrix used in most LRR methods, in MmCLRR, we adopt concept factorization to model the dictionary matrix. Concept factorization makes the dictionary update continuously during optimization, which enhances the completeness of dictionary and breaks through the bottleneck of using fixed dictionary matrix to describe subspace in LRR. In addition, we apply manifold regularization to further preserve the local topology of the data in the projecting. Benefiting by concept factorization and manifold regularization, the proposed method can capture the inherent subspace structure located in each view, and identify the latent subspace hidden in multi-view.
2. We apply MmCLRR to model cancer multi-omics data, and further propose a new cancer clustering framework based on multi omics data. This will make the clustering study of cancer get rid of the limitation of single omics data, and greatly promote the development of multi-omics data in cancer clustering research.
3. The clustering framework of MmCLRR is used to study cancer clustering, and many experiments of cancer samples clustering based on multi-omics data are provided. The experimental results indicate that it is feasible to cluster cancer using multi-omics data. These results also demonstrate the effectiveness of MmCLRR in cancer clustering.

The remainder of this article is schemed as follows. In Sect. 2 a brief overview of the related work including LRR, manifold regularization as well as concept factorization is given. In Sect. 3, the developed MmCLRR method and its model on cancer multi-omics data are elaborated. The experiment results and the performance analysis based on MmCLRR and several comparison methods are demonstrated in Sect. 4. The conclusion of this work is given in Sect. 5.

## Methods

### LRR and MLRSSC

LRR is an important method of subspace clustering firstly developed by Liu et al. [34]. The main idea of LRR is to regard high-dimensional data as a mapping from low-dimensional space. For specific high-dimensional data, the corresponding low-dimensional space is usually a combination of several independent subspaces. In other words, high-dimensional data can be regarded as the mapping combination of these low-dimensional subspaces. The tenet of LRR is to seek the subspace structure contained in high-dimensional observed data by calculating the mapping coefficient. Because the dimension of the low-dimensional subspace is far lower than that of the original observation data, the mapping coefficient of the high-dimensional data is low rank. Therefore, LRR aims to obtain the lowest rank coefficient matrix by optimizing the rank minimization problem. For observation data ${\mathbf{X}}$, the object of LRR is defined as follows.

1 $$\mathop {\min }\limits_{{\mathbf{Z}}} rank({\mathbf{Z}}),{\text{ }}s.t.\;{\text{ }}{\mathbf{X}} = {\mathbf{AZ}}.$$

Here, ${\mathbf{A}}$ is the projection basis from high-dimensional space to low-dimensional space, often known as dictionary. The high-dimensional observation data can be formed by a linear combination of ${\mathbf{A}}$, and the coefficients of linear combination constitutes matrix ${\mathbf{Z}}$. So ${\mathbf{Z}}$ is called coefficient matrix, also named as low-rank representation matrix or low-rank affinity matrix. Supposing ${\mathbf{Z}} = \left[ {{\mathbf{z}}_{1} {\mathbf{,z}}_{2} {\mathbf{,}}{\text{ }}...{\text{ }}{\mathbf{,z}}_{n} } \right]$, where $n$ is the number of data points, then the column vector ${\mathbf{z}}_{j}$ is also thought as the mapping representation of the original data points $j$ in each low-dimensional subspace. Therefore, matrix ${\mathbf{Z}}$ contains abundant subspace structure information for subspace segmentation.

In practice, the original high-dimensional data are directly regarded as ${\mathbf{A}}$. And the nuclear norm is used as the surrogate of rank function to obtain the convex optimization of problem (1). The deformation of the optimal problem of LRR is as follows.

2 $$\mathop {\min }\limits_{{\mathbf{Z}}} \left\| {\mathbf{Z}} \right\|_{*} ,{\text{ }}s.t.{\text{ }}\;{\mathbf{X}} = {\mathbf{XZ}}.$$

Here, $\left\| \cdot \right\|_{*}$ is the nuclear norm and $\left\| {\mathbf{Z}} \right\|_{*} {\text{ = }}\sum\limits_{i} {\sigma _{i} }$, where $\sigma _{i}$ is the singular value of ${\mathbf{Z}}$. At this point, the elements of ${\mathbf{Z}}$ can be regarded as the similar expression between the original data points in the mapping space. In subspace segmentation, the data points with high similarity expression are approximately from the same subspace, so these data points are clustered into the same class.

Generally, the observations from the real world are noisy. In order to reduce the influence of noise on subspace learning, an error item is usually added to the object of LRR. For random noise, we often employ $l_{1}$-norm to characterize the error term. To this end, the optimization problem (2) can be transformed as:

3 $$\mathop {\min }\limits_{{{\mathbf{Z}},{\mathbf{E}}}} \left\| {\mathbf{Z}} \right\|_{*} {\text{ + }}\alpha \left\| {\mathbf{E}} \right\|_{1} ,{\text{ }}s.t.\;{\text{ }}{\mathbf{X}} = {\mathbf{XZ}} + {\mathbf{E}},$$

where ${\mathbf{E}}$ indicates the error, $\left\| \cdot \right\|_{1}$ denotes $l_{1}$-norm which is a regularization strategy to make a matrix sparse and the $l_{1}$-norm of matrix ${\mathbf{E}}$ is defined as $\left\| {\mathbf{E}} \right\|_{1} {\text{ = }}\sum\limits_{i} {\sum\limits_{j} {\left| {e_{{ij}} } \right|} }$, $\alpha$ is a super parameter to balance the noise. After LRR decomposing, the minimizer ${\mathbf{E}}^{*}$ and ${\mathbf{Z}}^{*}$ can be acquired. Among them, ${\mathbf{E}}^{*}$ can be used for noise removal [35, 36] or feature selection, ${\mathbf{Z}}^{*}$ can be used for subspace clustering [37] or classification [38–40], and ${\mathbf{XZ}}^{{\text{*}}}$ can be used for the low-rank recovery of original data [41].

MLRSSC is a multi-view clustering framework. It jointly learns a subspace representation by constructing a consistent similarity matrix shared by multi-view data. Given a dataset ${\mathbf{X}} = \left\{ {{\mathbf{X}}^{{\left( 1 \right)}} ,{\mathbf{X}}^{{\left( 2 \right)}} , \cdots ,{\mathbf{X}}^{{\left( {m_{v} } \right)}} } \right\}$ containing $m_{v}$ views, ${\mathbf{X}}^{{\left( v \right)}} \in R^{{M^{{(v)}} \times N}}$ corresponds to view $v$. Here, $N$ denotes the number of samples, and all views are from the same sample group. $M^{{\left( v \right)}}$ denotes the feature number of view $v$, and each view has its own features. In MLRSSC, for the purpose of learning a joint representation matrix, the regularization item is introduced to ensure the agreement between affinity matrices of pairwise views. At the same time, MLRSSC encourages the sparsity of low-rank representation. The objective function of MLRSSC is as follows.

4 $$\begin{gathered} \min \sum\limits_{{v = 1}}^{{m_{v} }} {\left( {\beta _{1} \left\| {{\mathbf{C}}^{{\left( v \right)}} } \right\|_{*} + \beta _{2} \left\| {{\mathbf{C}}^{{\left( v \right)}} } \right\|_{1} } \right)} + \sum\limits_{{1 \le v,w \le m_{v} ,v \ne w}}^{{}} {\lambda ^{{\left( v \right)}} \left\| {{\mathbf{C}}^{{\left( v \right)}} - {\mathbf{C}}^{{\left( w \right)}} } \right\|_{F}^{2} } \hfill \\ \quad s.t.\;{\text{ }}{\mathbf{X}}^{{\left( v \right)}} = {\mathbf{X}}^{{\left( v \right)}} {\mathbf{C}}^{{\left( v \right)}} ,{\text{diag}}({\mathbf{C}}^{{\left( v \right)}} ) = 0,v = 1, \ldots ,m_{v} . \hfill \\ \end{gathered}$$

Here, ${\mathbf{C}}^{{\left( v \right)}}$ is the low-rank representation corresponding to view $v$. $\beta _{1}$, $\beta _{2}$ and $\lambda ^{{\left( v \right)}}$ are parameters to balance low rank, sparse constraints and the consistency across views, respectively.

### Manifold regularization

Usually, the naturally generated data are approximately regarded as to be located in a certain manifold. Many studies have shown that the manifold structure of data is very important to the low-dimensional space learning or low-dimensional representation [42, 43]. However, these data are usually from high-dimensional space and have insufficient sample size, which makes it very difficult to obtain the global structure of the data manifold accurately. In manifold theory, each small enough part of a manifold is considered to come from Euclidean space and the manifold can be regarded as the adhesion of these small parts. So, researchers focus on preserving the local structure information of manifold to learn the topological properties from scattered data. In practice, the nearest neighbor graph based on data points is used to model the local geometry of the data manifold [44]. Given ${\mathbf{X}} = \left[ {{\text{x}}_{1} {\mathbf{,x}}_{2} {\mathbf{,}}{\text{ }} \ldots {\text{ }}{\mathbf{,}}{\text{x}}_{n} } \right]$ from an underlying submanifold of high-dimensional space, $n$ is the number of data points, we can construct a nearest neighbor graph $\user2{G}$ with $n$ nodes. In $\user2{G}$, each node corresponds to a sample point, and the sample points are connected by edges. More specifically, we first determine the $k$-nearest neighbors of each data point by calculating the Euclidean distance between the data points, and then assign the weights of the connecting edges between the data points. There are three main ways to assign the weights of edges. For more details, please refer [45]. In this paper, we use Gaussian Kernel to calculate the weights. For the edge connecting data points ${\text{x}}_{i}$ and ${\text{x}}_{j}$, the according weight is set as

5 $$H_{{ij}} = \left\{ {\begin{array}{*{20}l} {{\text{e}}^{{ - \frac{{\left\| {{\mathbf{x}}_{i} - {\mathbf{x}}_{j} } \right\|^{2} }}{{2t^{2} }}}} } \hfill & {{\text{if}}\;{\mathbf{ x}}_{j} \in N_{k} \left( {{\mathbf{x}}_{i} } \right){\text{ or }}{\mathbf{x}}_{i} \in N_{k} \left( {{\mathbf{x}}_{j} } \right)} \hfill \\ 0 \hfill & {{\text{otherwise}}} \hfill \\ \end{array} } \right..$$

Here, $k$ is the number of nearest neighbors. $N_{k} \left( {{\mathbf{x}}_{j} } \right)$ denotes the set of k nearest neighbors based on ${\text{x}}_{j}$. For high-dimensional data ${\mathbf{X}}$, all the weights of the edges between data points form a symmetric weight matrix, which is denoted as ${\mathbf{H}}$. Because ${\mathbf{H}}$ contains the local structure information of the submanifold in which the observed data are located, based on ${\mathbf{H}}$, every data point of the observation data can be represented as a linear combination of its nearest neighbors.

According to the basic assumption of manifold theory, namely, if two data points in the data manifold are close to each other, their mappings of the two data points in a new coordinates are still close [46], we can minimize the objective as shown in formula (6) to preserve the inherent local structure of high-dimensional data.

6 $$\begin{aligned} & \sum\limits_{{i,j}} {\left\| {{\mathbf{z}}_{i} - {\mathbf{z}}_{j} } \right\|} ^{2} H_{{ij}} \\ & \quad {\text{ = }}\sum\limits_{i} {{\mathbf{z}}_{i} ^{T} {\mathbf{z}}_{i} D_{{ii}} } - \sum\limits_{{i,j}} {{\mathbf{z}}_{i} ^{T} {\mathbf{z}}_{j} H_{{ij}} } \\ & \quad = tr\left( {{\mathbf{Z}}\left( {{\mathbf{D}} - {\mathbf{H}}} \right){\mathbf{Z}}^{T} } \right) \\ & \quad = tr\left( {{\mathbf{ZLZ}}^{T} } \right). \\ \end{aligned}$$

Here, ${\mathbf{z}}_{i}$ is the mapping expression of data point ${\text{x}}_{i}$. The matrix ${\mathbf{D}}$ is diagonal, and its diagonal element is defined as $D_{{ii}} = \sum\nolimits_{j} {H_{{ji}} }$. ${\mathbf{L}}{\text{ = }}{\mathbf{D}} - {\mathbf{H}}$ is named as graph Laplacian matrix [47]. $tr( \cdot )$ denotes the trace function. The manifold regularization is widely used to enhance various algorithms [48–50].

### Concept factorization

The basic idea of concept factorization is that each prominent concept in the observation data set can be represented by associating data points with similar concepts [33]. Namely, each concept can be represented by the linear combination of the whole data points. The vectors generated by this linear combination characterizes the key concepts shared by relevant data points. Given data set ${\mathbf{X}} = \left[ {{\text{x}}_{1} {\mathbf{,x}}_{2} {\mathbf{,}}{\text{ }} \ldots {\text{ }}{\mathbf{,}}{\text{x}}_{n} } \right]$, ${\text{x}}_{i}$ denotes data point $i$, then the concept ${\mathbf{R}}_{c}$ can be represented mathematically as follows.

7 $$R_{c} {\text{ = }}\sum\limits_{{i = 1}}^{n} {w_{{ic}} {\mathbf{x}}_{i} } .$$

Here, $w_{{ic}}$ is an association coefficient, showing the degree of association of ${\text{x}}_{i}$ with concept ${\mathbf{R}}_{c}$.

On the other hand, the data point in the observation data can also be approximated by linear union of these concepts, in mathematics, which can be expressed in the following formula.

8 $${\mathbf{x}}_{i} {\text{ = }}\sum\limits_{c}^{{}} {m_{{ic}} {\mathbf{R}}_{c} } ,$$

where $m_{{ic}}$ is overlap coefficient that indicates how well ${\text{x}}_{i}$ overlaps the concept ${\mathbf{R}}_{c}$. We denote the association coefficient matrix composed of coefficient $w_{{ic}}$ as ${\mathbf{W}}$, and the overlap coefficient matrix formed by $m_{{ic}}$ as ${\mathbf{M}}$. In mathematics, the idea of concept factorization can be formulated as follows.

9 $${\mathbf{X}} \approx {\mathbf{XWM}}^{T} .$$

In Eq. (9), ${\mathbf{XW}}$ can be seen as center of concept, and ${\mathbf{M}}$ can be regarded as the projection of original data point on concept center. After concept factorization, we can find the prominent concepts in a given dataset and cluster membership for each data point. Due to the excellent performance of concept factorization in concept discovery, it has been widely concerned and applied into clustering research [51, 52].

### The proposed MmCLRR method

In this part, the proposed Multi-view Manifold Regularized Compact Low-Rank Representation (MmCLRR) method and its solution are elaborated. And then the model of MmCLRR based on cancer multi-omics data is given.

### Problem formulation and the solution

Most LRR-based methods select observed data as dictionary to learn the low-rank representation of high-dimensional data. The noise contained in the data and the insufficient sample size will lead to the incompleteness of the dictionary, which will directly affect the mapping expression of the original data in the low-dimensional space. To this end, we introduce concept decomposition into MLRSSC method to reconstruct dictionary matrix using the linear combination of original sample points. Meanwhile, in view of the advantages of manifold regularization in exploring the local structure of manifold, we further introduce manifold regularization into our method. In MmCLRR, we combine the sparse LRR model with the data dictionary modeling and manifold regularization constraints to obtain the subspace structure information comprehensively. Given a dataset with $m_{v}$ views ${\mathbf{X}} = \left\{ {{\mathbf{X}}^{{\left( 1 \right)}} ,{\mathbf{X}}^{{\left( 2 \right)}} , \ldots ,{\mathbf{X}}^{{\left( {m_{v} } \right)}} } \right\}$, where ${\mathbf{X}}^{{\left( v \right)}}$ represents the $v$-th feature view, the MmCLRR method can be formulated as

10 $$\begin{aligned} & \min \sum\limits_{{v = 1}}^{{m_{v} }} {\left[ {\left\| {{\mathbf{Z}}^{{\left( v \right)}} } \right\|_{*} + \gamma _{1} \left\| {{\mathbf{Z}}^{{\left( v \right)}} } \right\|_{1} + \gamma _{2} \left\| {{\mathbf{E}}^{{\left( v \right)}} } \right\|_{{2,1}} + \gamma _{3} tr\left( {{\mathbf{Z}}^{{^{{\left( v \right)}} }} {\mathbf{L}}^{{\left( v \right)}} {\mathbf{Z}}^{{\left( v \right)T}} } \right)} \right]} {\text{ }} + \sum\limits_{{1 \le v,w \le m_{v} ,v \ne w}}^{{}} {\gamma ^{{\left( v \right)}} \left\| {{\mathbf{Z}}^{{\left( v \right)}} - {\mathbf{Z}}^{{\left( w \right)}} } \right\|_{F}^{2} } \\ & \quad s.t.\;{\text{ }}{\mathbf{X}}^{{\left( v \right)}} = {\mathbf{X}}^{{\left( v \right)}} {\mathbf{W}}^{{\left( v \right)}} {\mathbf{Z}}^{{\left( v \right)}} + {\mathbf{E}}^{{\left( v \right)}} ,{\mathbf{W}}^{{\left( v \right)T}} {\mathbf{W}}^{{\left( v \right)}} = {\mathbf{I}}. \\ \end{aligned}$$

**Fig. 1:**
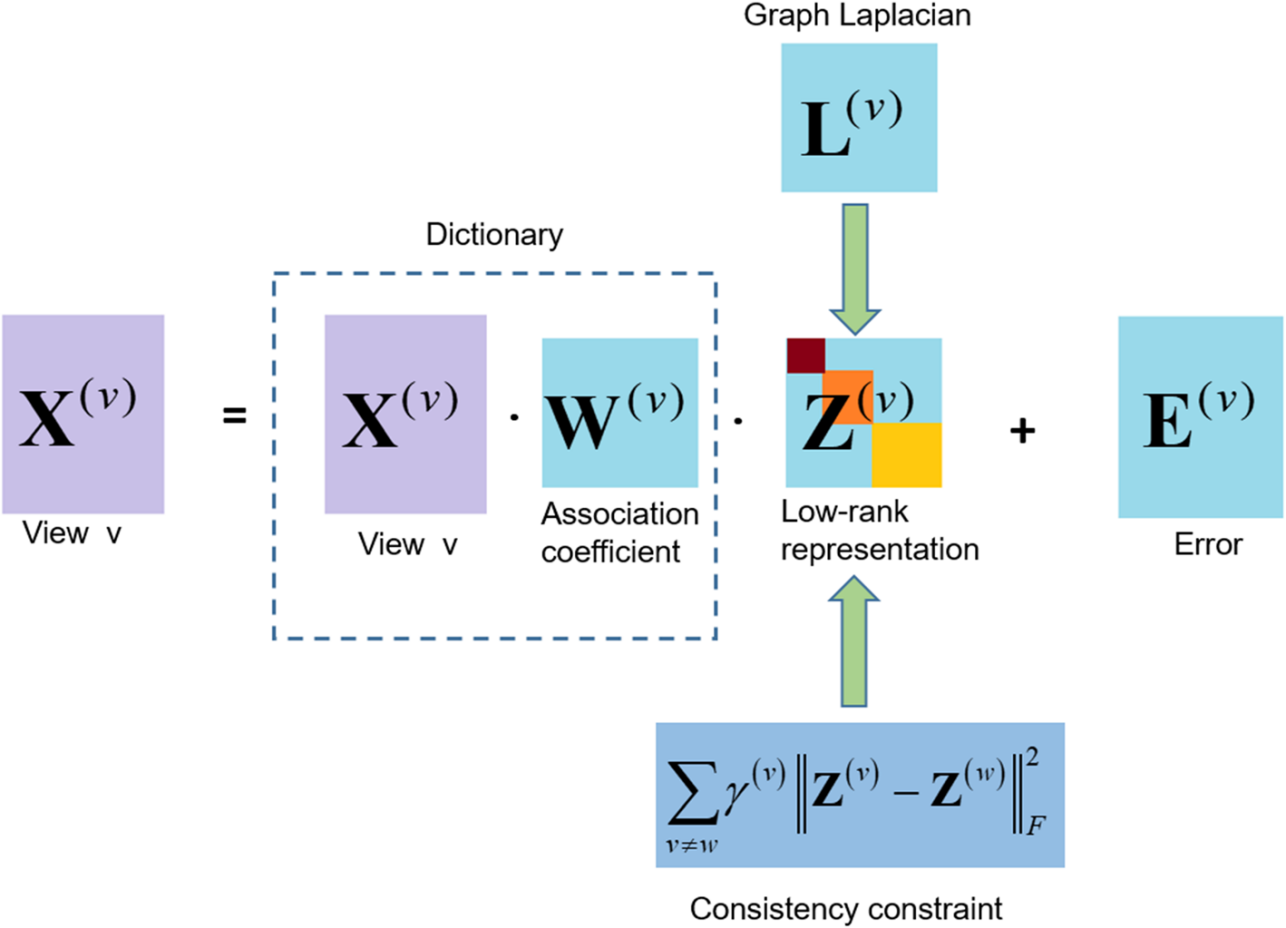
The decomposition flowchart of MmCLRR on view $v$

Here, ${\mathbf{Z}}^{{\left( v \right)}}$, ${\mathbf{E}}^{{\left( v \right)}}$ is the low-rank affinity matrix and error item corresponding to view ${\mathbf{X}}^{{\left( v \right)}}$. ${\mathbf{X}}^{{\left( v \right)}} {\mathbf{W}}^{{\left( v \right)}}$ represents the center of cluster of ${\mathbf{X}}^{{\left( v \right)}}$. ${\mathbf{W}}^{{\left( v \right)T}} {\mathbf{W}}^{{\left( v \right)}} = {\mathbf{I}}$ is a constraint to ensure the stability of the model. $\gamma _{1}$, $\gamma _{2}$ and $\gamma _{3}$ are penalty parameters. The parameter $\gamma ^{{\left( v \right)}}$ is to balance the consistency of coefficient matrix between different views. The last item in (10) can help to reduce the noise propagation in low-rank affinity matrix and encourage the similarity between the representation matrices of views. Take view $v$ as an example, the decomposition of MmCLRR is shown in Fig. 1.

We use Linearized Alternating Direction Method with Adaptive Penalty (LADMAP) [53] to solve the optimization problem in (10). In order to facilitate the solution, we introduce three auxiliary variables ${\mathbf{Z}}_{A}$, ${\mathbf{Z}}_{B}$ and ${\mathbf{Z}}_{C}$ into the objective of MmCLRR. The problem (10) is converted into

11 $$\begin{aligned} & \min \sum\limits_{{v = 1}}^{{m_{v} }} {\left[ {\left\| {{\mathbf{Z}}_{A}^{{\left( v \right)}} } \right\|_{*} + \gamma _{1} \left\| {{\mathbf{Z}}_{B}^{{\left( v \right)}} } \right\|_{1} + \gamma _{2} \left\| {{\mathbf{E}}^{{\left( v \right)}} } \right\|_{{2,1}} + \gamma _{3} tr\left( {{\mathbf{Z}}_{A}^{{\left( v \right)}} {\mathbf{L}}^{{\left( v \right)}} {\mathbf{Z}}_{A}^{{\left( v \right)T}} } \right)} \right]} {\text{ }} + \sum\limits_{{1 \le v,w \le m_{v} ,v \ne w}}^{{}} {\gamma ^{{\left( v \right)}} \left\| {{\mathbf{Z}}_{C}^{{\left( v \right)}} - {\mathbf{Z}}^{{\left( w \right)}} } \right\|_{F}^{2} } \\ & \quad {\text{ }}s.t.\;{\text{ }}{\mathbf{Z}}_{A}^{{\left( v \right)}} = {\mathbf{Z}}^{{\left( v \right)}} ,{\mathbf{Z}}_{B}^{{\left( v \right)}} = {\mathbf{Z}}^{{\left( v \right)}} ,{\mathbf{Z}}_{C}^{{\left( v \right)}} = {\mathbf{Z}}^{{\left( v \right)}} ,{\mathbf{H}}^{{\left( v \right)}} = {\mathbf{W}}^{{\left( v \right)}} {\mathbf{Z}}^{{\left( v \right)}} , \\ & \quad \quad {\text{ }}{\mathbf{X}}^{{\left( v \right)}} = {\mathbf{X}}^{{\left( v \right)}} {\mathbf{H}}^{{\left( v \right)}} + {\mathbf{E}}^{{\left( v \right)}} ,{\mathbf{W}}^{{\left( v \right)T}} {\mathbf{W}}^{{\left( v \right)}} = {\mathbf{I}}. \\ \end{aligned}$$

Then, we draw into augmented Lagrangian method. The function (11) is recast as

12 $$\begin{aligned} & \min \left\| {{\mathbf{Z}}_{A}^{{\left( v \right)}} } \right\|_{*} + \gamma _{1} \left\| {{\mathbf{Z}}_{B}^{{\left( v \right)}} } \right\|_{1} + \gamma _{2} \left\| {{\mathbf{E}}^{{\left( v \right)}} } \right\|_{{2,1}} + \gamma _{3} tr\left( {{\mathbf{Z}}_{A}^{{\left( v \right)}} {\mathbf{L}}^{{\left( v \right)}} {\mathbf{Z}}_{A}^{{\left( v \right)T}} } \right) + {\text{ }}\sum\limits_{{1 \le v,w \le m_{v} ,v \ne w}}^{{}} {\gamma ^{{\left( v \right)}} \left\| {{\mathbf{Z}}_{C}^{{\left( v \right)}} - {\mathbf{Z}}^{{\left( w \right)}} } \right\|} _{F}^{2} \\ & \quad \quad + \frac{{\mu _{1} }}{2}\left\| {{\mathbf{X}}^{{\left( v \right)}} - {\mathbf{X}}^{{\left( v \right)}} {\mathbf{H}}^{{\left( v \right)}} - {\mathbf{E}}^{{\left( v \right)}} + \frac{{{\mathbf{Y}}_{1} }}{{\mu _{1} }}} \right\|_{F}^{2} + \frac{{\mu _{2} }}{2}\left\| {{\mathbf{H}}^{{\left( v \right)}} - {\mathbf{W}}^{{\left( v \right)}} {\mathbf{Z}}^{{\left( v \right)}} + \frac{{{\mathbf{Y}}_{2} }}{{\mu _{2} }}} \right\|_{F}^{2} \\ & \quad \quad + \frac{{\mu _{3} }}{2}\left\| {{\mathbf{Z}}^{{\left( v \right)}} - {\mathbf{Z}}_{A}^{{\left( v \right)}} + \frac{{{\mathbf{Y}}_{3} }}{{\mu _{3} }}} \right\|_{F}^{2} + \frac{{\mu _{4} }}{2}\left\| {{\mathbf{Z}}^{{\left( v \right)}} - {\mathbf{Z}}_{B}^{{\left( v \right)}} + \frac{{{\mathbf{Y}}_{4} }}{{\mu _{4} }}} \right\|_{F}^{2} + \frac{{\mu _{5} }}{2}\left\| {{\mathbf{Z}}^{{\left( v \right)}} - {\mathbf{Z}}_{C}^{{\left( v \right)}} + \frac{{{\mathbf{Y}}_{5} }}{{\mu _{5} }}} \right\|_{F}^{2} \\ & \quad {\text{s}}.{\text{t}}{\text{. }}{\mathbf{W}}^{{\left( v \right)T}} {\mathbf{W}}^{{\left( v \right)}} = {\mathbf{I}}. \\ \end{aligned}$$

Here, $\mu _{1} {\text{ = }}\mu _{2} {\text{ = }}\mu _{3} {\text{ = }}\mu _{4} {\text{ = }}\mu _{5} {\text{ = }}\mu$ are penalty parameters, ${\mathbf{Y}}_{1} \sim {\mathbf{Y}}_{5}$ are Lagrange multipliers. Next,

The formula (12) is separated into the following sub problems with respect to ${\mathbf{Z}}_{A}^{{\left( v \right)}}$, ${\mathbf{Z}}_{B}^{{\left( v \right)}}$, ${\mathbf{Z}}_{C}^{{\left( v \right)}}$, ${\mathbf{Z}}^{{\left( v \right)}}$, ${\mathbf{H}}^{{\left( v \right)}}$, ${\mathbf{W}}^{{\left( v \right)}}$ and ${\mathbf{E}}_{{}}^{{\left( v \right)}}$.

13 $$\begin{aligned} l_{1} \left( {{\mathbf{Z}}^{{\left( v \right)}} } \right) & {\text{ = }}\mathop {\arg \min }\limits_{{\mathbf{Z}}} \frac{{\mu _{2} }}{2}\left\| {{\mathbf{H}}^{{\left( v \right)}} - {\mathbf{W}}^{{\left( v \right)}} {\mathbf{Z}}^{{\left( v \right)}} + \frac{{{\mathbf{Y}}_{2} }}{{\mu _{2} }}} \right\|_{F}^{2} + \frac{{\mu _{3} }}{2}\left\| {{\mathbf{Z}}^{{\left( v \right)}} - {\mathbf{Z}}_{A}^{{\left( v \right)}} + \frac{{{\mathbf{Y}}_{3} }}{{\mu _{3} }}} \right\|_{F}^{2} \\ & \quad + \frac{{\mu _{4} }}{2}\left\| {{\mathbf{Z}}^{{\left( v \right)}} - {\mathbf{Z}}_{B}^{{\left( v \right)}} + \frac{{{\mathbf{Y}}_{4} }}{{\mu _{4} }}} \right\|_{F}^{2} + \frac{{\mu _{5} }}{2}\left\| {{\mathbf{Z}}^{{\left( v \right)}} - {\mathbf{Z}}_{C}^{{\left( v \right)}} + \frac{{{\mathbf{Y}}_{5} }}{{\mu _{5} }}} \right\|_{F}^{2} . \\ \end{aligned}$$

14 $$l_{2} \left( {{\mathbf{Z}}_{A}^{{\left( v \right)}} } \right){\text{ = }}\mathop {\arg \min }\limits_{{{\mathbf{Z}}_{A} }} \left\| {{\mathbf{Z}}_{A}^{{\left( v \right)}} } \right\|_{*} + \gamma _{3} tr\left( {{\mathbf{Z}}_{A}^{{\left( v \right)}} {\mathbf{L}}^{{\left( v \right)}} {\mathbf{Z}}_{A}^{{\left( v \right)T}} } \right) + \frac{{\mu _{3} }}{2}\left\| {{\mathbf{Z}}^{{\left( v \right)}} - {\mathbf{Z}}_{A}^{{\left( v \right)}} + \frac{{{\mathbf{Y}}_{3} }}{{\mu _{3} }}} \right\|_{F}^{2} .$$

15 $$l_{3} \left( {{\mathbf{Z}}_{B}^{{\left( v \right)}} } \right){\text{ = }}\mathop {\arg \min }\limits_{{{\mathbf{Z}}_{B} }} \gamma _{1} \left\| {{\mathbf{Z}}_{B}^{{\left( v \right)}} } \right\|_{1} + \frac{{\mu _{4} }}{2}\left\| {{\mathbf{Z}}^{{\left( v \right)}} - {\mathbf{Z}}_{B}^{{\left( v \right)}} + \frac{{{\mathbf{Y}}_{4} }}{{\mu _{4} }}} \right\|_{F}^{2} .$$

16 $$l_{4} \left( {{\mathbf{Z}}_{C}^{{\left( v \right)}} } \right){\text{ = }}\mathop {\arg \min }\limits_{{{\mathbf{Z}}_{C} }} {\text{ }}\sum\limits_{{1 \le v,w \le m_{v} ,v \ne w}}^{{}} {\gamma ^{{\left( v \right)}} \left\| {{\mathbf{Z}}_{C}^{{\left( v \right)}} - {\mathbf{Z}}^{{\left( w \right)}} } \right\|} _{F}^{2} + \frac{{\mu _{5} }}{2}\left\| {{\mathbf{Z}}^{{\left( v \right)}} - {\mathbf{Z}}_{C}^{{\left( v \right)}} + \frac{{{\mathbf{Y}}_{5} }}{{\mu _{5} }}} \right\|_{F}^{2} .$$

17 $$l_{5} \left( {{\mathbf{E}}^{{\left( v \right)}} } \right){\text{ = }}\mathop {{\text{arg min}}}\limits_{{\mathbf{E}}} \gamma _{2} \left\| {{\mathbf{E}}^{{\left( v \right)}} } \right\|_{{2,1}} + \frac{{\mu _{1} }}{2}\left\| {{\mathbf{X}}^{{\left( v \right)}} - {\mathbf{X}}^{{\left( v \right)}} {\mathbf{H}}^{{\left( v \right)}} - {\mathbf{E}}^{{\left( v \right)}} + \frac{{{\mathbf{Y}}_{1} }}{{\mu _{1} }}} \right\|_{F}^{2} .$$

18 $$l_{6} \left( {{\mathbf{H}}^{{\left( v \right)}} } \right){\text{ = }}\mathop {\arg \min }\limits_{{\mathbf{H}}} \frac{{\mu _{1} }}{2}\left\| {{\mathbf{X}}^{{\left( v \right)}} - {\mathbf{X}}^{{\left( v \right)}} {\mathbf{H}}^{{\left( v \right)}} - {\mathbf{E}}^{{\left( v \right)}} + \frac{{{\mathbf{Y}}_{1} }}{{\mu _{1} }}} \right\|_{F}^{2} + \frac{{\mu _{2} }}{2}\left\| {{\mathbf{H}}^{{\left( v \right)}} - {\mathbf{W}}^{{\left( v \right)}} {\mathbf{Z}}^{{\left( v \right)}} + \frac{{{\mathbf{Y}}_{2} }}{{\mu _{2} }}} \right\|_{F}^{2} .$$

19 $$l_{7} \left( {{\mathbf{W}}^{{\left( v \right)}} } \right){\text{ = }}\mathop {\arg \min }\limits_{{\mathbf{W}}} \frac{{\mu _{2} }}{2}\left\| {{\mathbf{H}}^{{\left( v \right)}} - {\mathbf{W}}^{{\left( v \right)}} {\mathbf{Z}}^{{\left( v \right)}} + \frac{{{\mathbf{Y}}_{2} }}{{\mu _{2} }}} \right\|_{F}^{2} {\text{ s}}{\text{.t}}{\text{. }}{\mathbf{W}}^{{\left( v \right)T}} {\mathbf{W}}^{{\left( v \right)}} = {\mathbf{I}}.$$

Then, the final iterative algorithm is obtained by solving the above sub problems in turn. It is assumed that all variables after the $k$-th iteration are known. For example, the variable.

${\mathbf{E}}_{{}}^{{\left( v \right)}}$ in the $k$-th iteration is marked as ${\mathbf{E}}_{k}^{{\left( v \right)}}$. The iteration rules for each variable are as follows.

(1) Updating ${\mathbf{Z}}^{{\left( v \right)}}$. According to sub problem (13), we take the derivative with respect to ${\mathbf{Z}}^{{\left( v \right)}}$ and let the derivative be equal to 0. Then the iteration rule of ${\mathbf{Z}}^{{\left( v \right)}}$ is obtained as follows.

20 $${\mathbf{Z}}_{{\left( {k{\text{ + }}1} \right)}}^{{\left( v \right)}} {\text{ = }}\left[ {\mu _{{2\left( k \right)}} {\mathbf{W}}_{{\left( k \right)}}^{{\left( v \right)T}} {\mathbf{W}}_{{\left( k \right)}}^{{\left( v \right)}} {\text{ + }}\left( {\mu _{{3\left( k \right)}} {\text{ + }}\mu _{{4\left( k \right)}} {\text{ + }}\mu _{{5\left( k \right)}} } \right){\mathbf{I}}} \right]^{{ - 1}} \times \left[ {\mu _{{2\left( k \right)}} {\mathbf{W}}_{{\left( k \right)}}^{{\left( v \right)T}} {\mathbf{H}}_{{\left( k \right)}}^{{\left( v \right)}} + \mu _{{3\left( k \right)}} {\mathbf{Z}}_{{A\left( k \right)}}^{{\left( v \right)}} } \right. + \mu _{{4\left( k \right)}} {\mathbf{Z}}_{{B\left( k \right)}}^{{\left( v \right)}} + \mu _{{5\left( k \right)}} {\mathbf{Z}}_{{C\left( k \right)}}^{{\left( v \right)}} \left. { + {\mathbf{W}}_{{\left( k \right)}}^{{\left( v \right)T}} {\mathbf{Y}}_{{2\left( k \right)}} - {\mathbf{Y}}_{{3\left( k \right)}} - {\mathbf{Y}}_{{4\left( k \right)}} - {\mathbf{Y}}_{{5\left( k \right)}} } \right].$$

(2) Updating ${\mathbf{Z}}_{A}^{{\left( v \right)}}$. We take the derivative of the problem (14) with regard to ${\mathbf{Z}}_{A}^{{\left( v \right)}}$, and denote the derivative as $\nabla _{{\mathbf{Z}}} f\left( {{\mathbf{Z}}_{{A\left( k \right)}}^{{\left( v \right)}} } \right)$.

$$\nabla _{{\mathbf{Z}}} f\left( {{\mathbf{Z}}_{{A\left( k \right)}}^{{\left( v \right)}} } \right) = \gamma _{3} \left[ {{\mathbf{Z}}_{{A\left( k \right)}}^{{\left( v \right)}} {\mathbf{L}}^{{\left( v \right)T}} + {\mathbf{Z}}_{{A\left( k \right)}}^{{\left( v \right)}} {\mathbf{L}}^{{\left( v \right)}} } \right] + \mu _{{3\left( k \right)}} \left( {{\mathbf{Z}}_{{A\left( k \right)}}^{{\left( v \right)}} - {\mathbf{Z}}_{{\left( k \right)}}^{{\left( v \right)}} - \frac{{{\mathbf{Y}}_{{3\left( k \right)}} }}{{\mu _{{3\left( k \right)}} }}} \right).$$

According to LADMAP, the solution of ${\mathbf{Z}}_{A}^{{\left( v \right)}}$ is transformed into the optimization of problem (22).

22 $$\mathop {\min }\limits_{{{\mathbf{Z}}_{A} }} \left\| {{\mathbf{Z}}_{A}^{{\left( v \right)}} } \right\|_{*} + \left\langle {\nabla _{{\mathbf{Z}}} f\left( {{\mathbf{Z}}_{{A\left( k \right)}}^{{\left( v \right)}} } \right),{\mathbf{Z}}_{A}^{{\left( v \right)}} - {\mathbf{Z}}_{{A\left( k \right)}}^{{\left( v \right)}} } \right\rangle + \frac{\eta }{2}\left\| {{\mathbf{Z}}_{A}^{{\left( v \right)}} - {\mathbf{Z}}_{{A\left( k \right)}}^{{\left( v \right)}} } \right\|_{F}^{2} ,$$

where $\eta = 2\gamma _{3} \left\| {{\mathbf{L}}^{{\left( v \right)}} } \right\|_{2} + \mu _{3} \left( {1 + \left\| {{\mathbf{X}}^{{\left( v \right)}} } \right\|_{2}^{2} } \right)$. Then the solution to problem (14) is as follows.

23 $${\mathbf{Z}}_{{A\left( {k + 1} \right)}}^{{\left( v \right)}} = \Theta _{{\frac{1}{\eta }}} \left( {{\mathbf{Z}}_{{A\left( k \right)}}^{{\left( v \right)}} - {{\nabla _{{\mathbf{Z}}} f\left( {{\mathbf{Z}}_{{A\left( k \right)}}^{{\left( v \right)}} } \right)} \mathord{\left/ {\vphantom {{\nabla _{{\mathbf{Z}}} f\left( {{\mathbf{Z}}_{{A\left( k \right)}}^{{\left( v \right)}} } \right)} \eta }} \right. \kern-\nulldelimiterspace} \eta }} \right).$$

Here,$\Theta \left( \cdot \right)$ denotes skinny singular value decomposition and $\Theta _{\varepsilon } \left( {\mathbf{A}} \right) = US_{\varepsilon } \left( \sum \right)V^{T}$, where $S_{\varepsilon } \left( x \right) = \text{sgn} \left( x \right)\max \left( {\left| x \right| - \varepsilon ,0} \right)$.

(3) Updating ${\mathbf{Z}}_{B}^{{\left( v \right)}}$. We find the partial derivative of problem (15) as below.

24 $$\frac{{\partial l_{3} }}{{\partial {\mathbf{Z}}_{{B\left( k \right)}}^{{\left( v \right)}} }}{\text{ = }}\mu _{{4\left( k \right)}} \left( {{\mathbf{Z}}_{{B\left( k \right)}}^{{\left( v \right)}} - {\mathbf{Z}}_{{\left( k \right)}}^{{\left( v \right)}} - {{{\mathbf{Y}}_{{4\left( k \right)}} } \mathord{\left/ {\vphantom {{{\mathbf{Y}}_{{4\left( k \right)}} } {\mu _{{4\left( k \right)}} }}} \right. \kern-\nulldelimiterspace} {\mu _{{4\left( k \right)}} }}} \right).$$

Let formula (24) be 0, and the expression of ${\mathbf{Z}}_{{B\left( k \right)}}^{{\left( v \right)}}$ is

25 $${\mathbf{Z}}_{{B\left( k \right)}}^{{\left( v \right)}} {\text{ = }}{\mathbf{Z}}_{{\left( k \right)}}^{{\left( v \right)}} + {{{\mathbf{Y}}_{{4\left( k \right)}} } \mathord{\left/ {\vphantom {{{\mathbf{Y}}_{{4\left( k \right)}} } {\mu _{{4\left( k \right)}} }}} \right. \kern-\nulldelimiterspace} {\mu _{{4\left( k \right)}} }}.$$

According to literature [54], the literation rule of ${\mathbf{Z}}_{B}^{{\left( v \right)}}$ is as follows.

26 $${\mathbf{Z}}_{{B\left( {k{\text{ + }}1} \right)}}^{{\left( v \right)}} {\text{ = }}S_{{\frac{{\gamma _{1} }}{{\mu _{{4\left( k \right)}} }}}} \left( {{\mathbf{Z}}_{{\left( k \right)}}^{{\left( v \right)}} + {{{\mathbf{Y}}_{{4\left( k \right)}} } \mathord{\left/ {\vphantom {{{\mathbf{Y}}_{{4\left( k \right)}} } {\mu _{{4\left( k \right)}} }}} \right. \kern-\nulldelimiterspace} {\mu _{{4\left( k \right)}} }}} \right)$$

(4) Updating ${\mathbf{Z}}_{C}^{{\left( v \right)}}$. Similar with ${\mathbf{Z}}_{A}^{{\left( v \right)}}$, the solution of problem (16) is as bellow.

27 $${\mathbf{Z}}_{{C\left( {k{\text{ + }}1} \right)}}^{{\left( v \right)}} {\text{ = }}\left[ {2\gamma ^{{\left( v \right)}} \left( {n_{v} - 1} \right){\text{ + }}\mu _{{5\left( k \right)}} } \right]^{{{\text{ - }}1}} \left( {2\gamma ^{{\left( v \right)}} \sum\limits_{{1 \le v,w \le m_{v} ,v \ne w}}^{{m_{v} = 3}} {{\mathbf{Z}}_{{\left( k \right)}}^{{\left( w \right)}} + \mu _{{5\left( k \right)}} {\mathbf{Z}}_{{\left( k \right)}}^{{\left( v \right)}} {\text{ + }}{\mathbf{Y}}_{{5\left( k \right)}} } } \right).$$

(5) Updating ${\mathbf{E}}_{{}}^{{\left( v \right)}}$. According to reference [34], the iterative formula of ${\mathbf{E}}_{{}}^{{\left( v \right)}}$ is.

28 $${\mathbf{E}}_{{\left( {k + 1} \right)}}^{{\left( v \right)}} \left( {:,i} \right) = \left\{ {\begin{array}{*{20}l} {\frac{{\left\| {{\mathbf{G}}\left( {:,i} \right)} \right\| - {{\gamma _{2} } \mathord{\left/ {\vphantom {{\gamma _{2} } {\mu _{{1\left( k \right)}} }}} \right. \kern-\nulldelimiterspace} {\mu _{{1\left( k \right)}} }}}}{{\left\| {{\mathbf{G}}\left( {:,i} \right)} \right\|}}{\mathbf{G}}\left( {:,i} \right),} \hfill & {{{\lambda _{2} } \mathord{\left/ {\vphantom {{\lambda _{2} } {\mu _{{1\left( k \right)}} }}} \right. \kern-\nulldelimiterspace} {\mu _{{1\left( k \right)}} }} < \left\| {{\mathbf{G}}\left( {:,i} \right)} \right\|} \hfill \\ 0 \hfill & {{\text{ otherwise}}} \hfill \\ \end{array} } \right..$$

Here, $${\mathbf{G}} = {\mathbf{X}}^{{\left( v \right)}} - {\mathbf{X}}^{{\left( v \right)}} {\mathbf{H}}_{{\left( k \right)}}^{{\left( v \right)}} + {{{\mathbf{Y}}_{{1\left( k \right)}} } \mathord{\left/ {\vphantom {{{\mathbf{Y}}_{{1\left( k \right)}} } {\mu _{{1\left( k \right)}} }}} \right. \kern-\nulldelimiterspace} {\mu _{{1\left( k \right)}} }}$$.

(6) Updating ${\mathbf{H}}_{{}}^{{\left( v \right)}}$. Similar with ${\mathbf{Z}}_{A}^{{\left( v \right)}}$ and ${\mathbf{Z}}_{C}^{{\left( v \right)}}$, the updating rule of ${\mathbf{H}}_{{}}^{{\left( v \right)}}$ is as.

29 $${\mathbf{H}}_{{\left( {k{\text{ + }}1} \right)}}^{{\left( v \right)}} = \left( {\mu _{{1\left( k \right)}} {\mathbf{X}}^{{\left( v \right)T}} {\mathbf{X}}^{{\left( v \right)}} + \mu _{{2\left( k \right)}} {\mathbf{I}}} \right)^{{ - 1}} \left( {\mu _{{1\left( k \right)}} {\mathbf{X}}^{{\left( v \right)T}} {\mathbf{X}}^{{\left( v \right)}} - \mu _{{1\left( k \right)}} {\mathbf{X}}^{{\left( v \right)T}} {\mathbf{E}}_{{\left( k \right)}}^{{\left( v \right)}} } \right. + {\mathbf{X}}^{{\left( v \right)T}} {\mathbf{Y}}_{{1\left( k \right)}} + \mu _{{2\left( k \right)}} \left. {{\mathbf{W}}_{{\left( k \right)}}^{{\left( v \right)}} {\mathbf{Z}}_{{\left( k \right)}}^{{\left( v \right)}} - {\mathbf{Y}}_{{2\left( k \right)}} } \right).$$

(7) Updating ${\mathbf{W}}_{{}}^{{\left( v \right)}}$. Referring to Theorem 1 in [55], we solve sub problem (19) and get the iteration of ${\mathbf{W}}_{{}}^{{\left( v \right)}}$ as follows.

30 $${\mathbf{W}}_{{\left( {k + 1} \right)}}^{{\left( v \right)}} = {\mathbf{UV}}^{T} .$$

Here, $$\left( {{\mathbf{H}}^{{\left( v \right)}} + {{{\mathbf{Y}}_{{2\left( k \right)}} } \mathord{\left/ {\vphantom {{{\mathbf{Y}}_{{2\left( k \right)}} } {\mu _{{2\left( k \right)}} }}} \right. \kern-\nulldelimiterspace} {\mu _{{2\left( k \right)}} }}} \right)\left( {{\mathbf{Z}}_{{\left( k \right)}}^{{\left( v \right)}} } \right)^{T} = {\mathbf{U}}\mho {\mathbf{V}}^{T} ,{\text{ }}\mho = {\text{diag}}\left( \delta \right)$$.

(8) Updating $${\mathbf{Y}}_{1} \sim {\mathbf{Y}}_{5}$$.

31 $${\mathbf{Y}}_{{1\left( {k + 1} \right)}} = {\mathbf{Y}}_{{1\left( k \right)}} + \mu _{1} \left( {{\mathbf{X}}^{{\left( v \right)}} - {\mathbf{X}}^{{\left( v \right)}} {\mathbf{H}}_{{\left( k \right)}}^{{\left( v \right)}} - {\mathbf{E}}_{{\left( k \right)}}^{{\left( v \right)}} } \right).$$

32 $${\mathbf{Y}}_{{2\left( {k + 1} \right)}} = {\mathbf{Y}}_{{2\left( k \right)}} + \mu _{2} \left( {{\mathbf{H}}_{{\left( k \right)}}^{{\left( v \right)}} - {\mathbf{W}}_{{\left( k \right)}}^{{\left( v \right)}} {\mathbf{Z}}_{{\left( k \right)}}^{{\left( v \right)}} } \right).$$

33 $${\mathbf{Y}}_{{3\left( {k + 1} \right)}} = {\mathbf{Y}}_{{3\left( k \right)}} + \mu _{3} \left( {{\mathbf{Z}}_{{\left( k \right)}}^{{\left( v \right)}} - {\mathbf{Z}}_{{A\left( k \right)}}^{{\left( v \right)}} } \right).$$

34 $${\mathbf{Y}}_{{4\left( {k + 1} \right)}} = {\mathbf{Y}}_{{4\left( k \right)}} + \mu _{4} \left( {{\mathbf{Z}}_{{\left( k \right)}}^{{\left( v \right)}} - {\mathbf{Z}}_{{B\left( k \right)}}^{{\left( v \right)}} } \right).$$

35 $${\mathbf{Y}}_{{5\left( {k + 1} \right)}} = {\mathbf{Y}}_{{5\left( k \right)}} + \mu _{5} \left( {{\mathbf{Z}}_{{\left( k \right)}}^{{\left( v \right)}} - {\mathbf{Z}}_{{C\left( k \right)}}^{{\left( v \right)}} } \right).$$

Finally, based on the low-rank representation matrix of each view, we calculate the fused affinity matrix ${\mathbf{Z}}^{*}$ by formula (36).

36 $${\mathbf{Z}}^{*} = \frac{{{\text{sum}}\left\{ {{\mathbf{Z}}^{{\left( 1 \right)}} ,{\mathbf{Z}}^{{\left( 2 \right)}} ,{\mathbf{Z}}^{{\left( 3 \right)}} } \right\}}}{{m_{v} }}.$$

The detailed optimization process of MmCLRR method is shown in Algorithm 1.
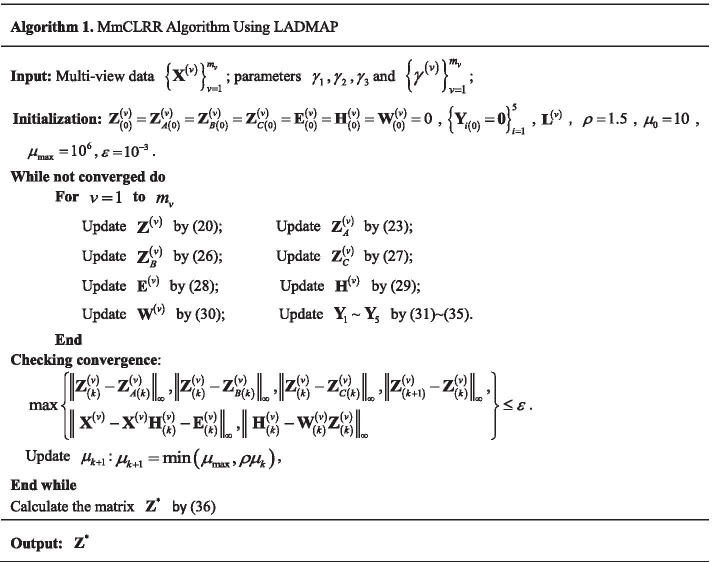


### The MmCLRR model on cancer multi-omics data

**Fig. 2:**
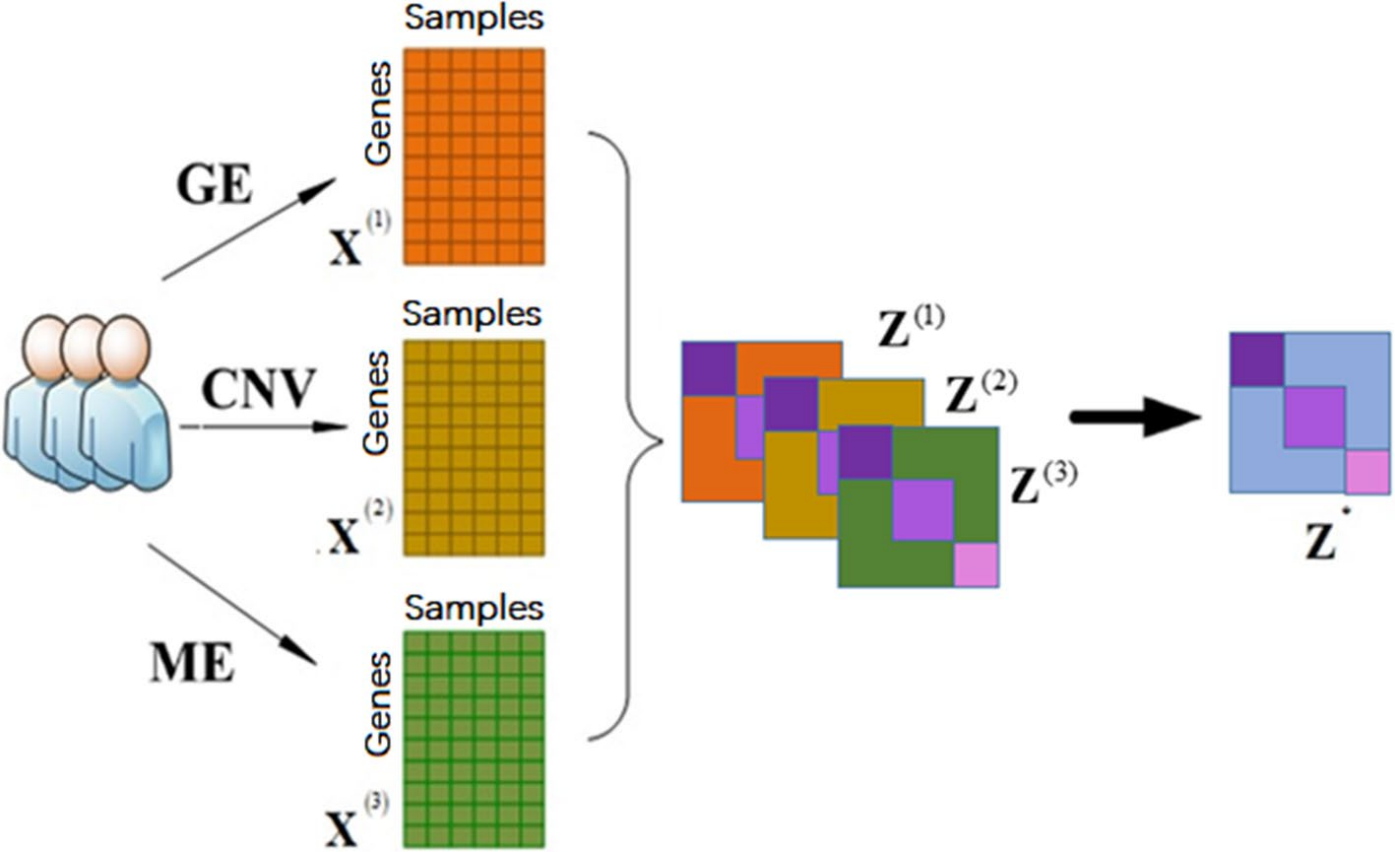
The MmCLRR model on cancer multi-omics data for cancer samples clustering

As mentioned earlier, besides gene expression data, DNA methylation and copy number variation also play important roles in the formation and development of cancer. And these omics data of cancer have been used alone or in combination with other data for cancer type research. This fully shows that these data contain the characteristic information needed in cancer type recognition. Thinking different omics data as the expression of cancer features at different levels, it is reasonable for us to regard that the feature information in these omics data can complement each other. Therefore, we intend to fuse the characteristic information of gene expression data, DNA methylation data and copy number variation data to cluster cancer samples. Here, we think of each omics data as a feature view of cancer, and use MmCLRR method to model these omics data. The schematic diagram of MmCLRR model on multi-omics data is shown in Fig. 2. In Fig. 2, gene expression data is abbreviated as GE, copy number variation is abbreviated as CNV, and DNA methylation is abbreviated as ME. ${\mathbf{Z}}_{{}}^{{\left( 1 \right)}} ,{\mathbf{Z}}_{{}}^{{\left( 2 \right)}}$ and ${\mathbf{Z}}_{{}}^{{\left( 3 \right)}}$ denote the low-rank representation matrix corresponding to GE, CNV and ME, respectively. In this model, we are not sure which omics data are more important, so we regard the proportion of each omics data in the model as the same, and use the same $\gamma ^{{\left( v \right)}}$ for all omics data. After the decomposition of MmCLRR, we adopt NCuts clustering method to cluster cancer samples based on the fused matrix ${\mathbf{Z}}^{*}$.

## Results

### Evaluation metrics

We use Accuracy (Acc) [56], Rand Index (RI) [57] and F1 measurment (F1) [58] as the evaluation metrics of clustering performance. The following is a brief introduction to these metrics.

Acc evaluates the clustering performance at the global level by calculating the matching degree between the experimental labels and the actual labels. It is defined as follows.

37 $${\text{Acc}} = \frac{{\sum\nolimits_{{i = 1}}^{N} {\delta \left( {p_{i} ,map\left( {q_{i} } \right)} \right)} }}{N} \times 100{\text{\% }}.$$

Here, $q_{i}$ and $p_{i}$ denote the experimental label and actual label of data point $i$, respectively. $N$ represents the number of data points. $map\left( {q_{i} } \right)$ is a function to match the experimental labels with the actual labels, and the method called Kuhn–Munkres [59] is usually employed to implement the matching. $\delta \left( {p_{i} ,map\left( {q_{i} } \right)} \right)$ is a function that compares the experimental tag with the actual tag. For data point $i$, if the experimental label $q_{i}$ is the same as the actual label $p_{i}$, the function value is assigned as 1, otherwise it is assigned as 0.

RI assesses the performance of clustering algorithm by comparing the relationship between the actual classification and the experimental classification. The following is the definition of RI.

38 $${\text{RI}} = \frac{{a + b}}{{C_{{n_{{samples}} }}^{2} }} \times 100{\text{\% }}.$$

Here, $a$ represents the number of data point pairs belonging to the same class in the actual classification and experimental classification. And $b$ denotes the number of data point pairs that are not in the same class. $C_{{n_{{samples}} }}^{2}$ is the total number of data pairs clustered or classified.

F1 is the average of precision rate and recall rate, which is defined as below.

39 $${\text{F1}} = \frac{{2*P*R}}{{P + R}} \times 100{\text{\% }}.$$

Here, $P = \frac{{TP}}{{TP + FP}} \times 100{\text{\% }}$ and $R{\text{ = }}\frac{{TP}}{{TP + FN}} \times 100{\text{\% }}$ denote precision rate and recall rate respectively, where $TP$ means that positive samples are clustered into positive class, $FP$ indicates that negative samples are wrongly classified into positive class, and $FN$ means that positive samples are classified into negative classes.

### Data sets

The data sets used in our study, including Head and Neck cancer (HNSC), Esophagus Cancer (ESCA) and Colon Adenocarcinoma (COAD), are downloaded from TCGA. Each data set contains three types of omics data, namely gene expression, DNA methylation data and copy number variation. And these omics data in each dataset come from the same batch of samples. Each of the three data sets includes cancer samples and normal samples. Specifically, HNSC consists of 398 cancer samples and 20 normal samples, ESCA includes 183 cancer samples and 9 normal samples, and COAD has got 262 cancer samples and 19 normal samples. The number of genes in gene expression, DNA methylation data and copy number variation data is 20502, 23,627 and 21,031, respectively. The samples and genes distribution of each omics data is shown in Table 1.

**Table 1:** Samples and genes distribution of each omics data in the experimental datasets

Datasets	Omics data	Genes	Normal samples	Cancer samples
HNSC	Gene expression	2002	20	398
	DNA methylation	23627	20	398
	Copy number variation	21031	20	398
COAD	Gene expression	20502	19	262
	DNA methylation	23627	19	262
	Copy number variation	21031	19	262
ESCA	Gene expression	20502	9	183
	DNA methylation	23627	9	183
	Copy number variation	21031	9	183

## Results and analysis

In order to test and verify the performance of our method in cancer samples clustering, we compare MmCLRR with the existing multi-views analysis methods, including ioNMF [24], SNF [23], Block- constraint Laplacian regularized LRR (BLLRR) [60] and MLRSSC [32]. In order to evaluate the performance of each clustering method more objectively, the clustering experiment of each method is executed 50 times, and the average values obtained from 50 experiments are used to evaluate the clustering results. The experimental results on HNSC, COAD and ESCA are shown in Table 2. And the best results of each data set are represented in bold. From Table 2, we can see that our method outstrip all comparison methods. Next, we will compare and analyze the experimental results in detail.

**Table 2:** The clustering performance of five methods on three experimental data sets

Multi-omics data	Metrics	ioNMF (%)	SNF (%)	BLLRR (%)	MLRSSC (%)	MmCLRR (%)
HNSC	Acc	69.38	90.29	97.58	78.71	**99.52**
	RI	57.41	83.74	92.00	67.23	**99.02**
	F1	44.45	47.51	74.22	55.37	**97.24**
COAD	Acc	64.76	86.30	98.27	74.93	**98.93**
	RI	54.87	78.53	89.38	63.21	**97.88**
	F1	66.06	50.29	72.13	58.90	**96.05**
ESCA	Acc	67.32	84.31	96.88	69.53	**96.25**
	RI	55.84	77.80	**93.91**	65.71	92.83
	F1	45.18	46.97	79.44	50.03	**83.50**

Best clustering results are highlighted in bold

Among the methods, BLLRR, MLRSSC and MmCLRR are low-rank subspace clustering (LRSC) methods. These LRSC methods mainly use the nuclear norm constraint to obtain the low-rank representation of multi-omics data, so as to explore the subspace structure of data. And, they construct the affinity matrix based on low-rank representation for cancer samples clustering. SNF is a network-based approach. It constructs similarity network for each omics data, and then integrates these networks generated by different omics data to realize samples clustering. The ioNMF approach is a NMF-based method. In ioNMF, different omics data are decomposed into a common fusion matrix and multiple independent sub matrixes at the same time, and then the common matrix is used to cluster samples. So, we firstly compare the three subspace clustering methods with ioNMF and SNF. From Table 2, we can find that the clustering results of the three subspace clustering methods are generally better than those of ioNMF and SNF. For this reason, we further calculate the mean values of BLLRR, MLRSSC and MmCLRR on each clustering metric (see Table 3). In Table 3, the average of LRSC methods is denoted as AVG-LRSC. And we also show the best results in bold. As can be seen from Table 3, the average clustering performance of these LRSC methods is significantly higher than the other two methods. The above analysis shows that LRSC method has a significant advantage in subspace learning.

**Table 3:** The average clustering results of three low-rank methods

Multi-omics data	Metrics	ioNMF (%)	SNF (%)	AVG-LRSC (%)
HNSC	Acc	69.38	90.29	**91.94**
	RI	57.41	83.74	**86.08**
	F1	44.45	47.51	**75.61**
COAD	Acc	64.76	86.30	**90.71**
	RI	54.87	78.53	**83.49**
	F1	66.06	50.29	**75.69**
ESCA	Acc	67.32	84.31	**87.55**
	RI	55.84	77.80	**84.15**
	F1	45.18	46.97	**70.99**

Best clustering results are highlighted in bold

**Fig. 3:**
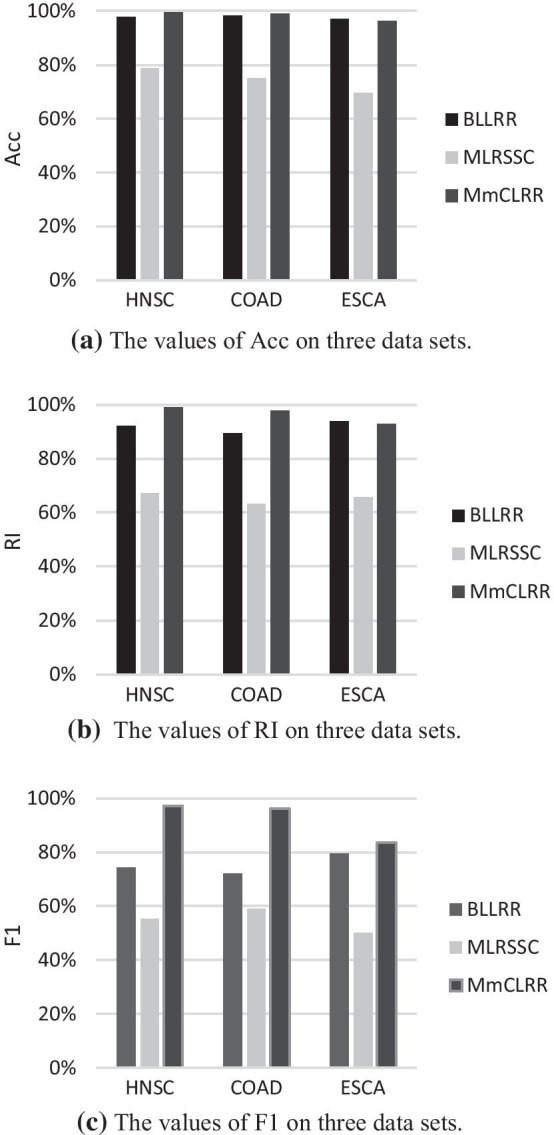
The clustering results of three LRSC methods

Among the three LRSC methods, MLRSSC method does not take the local topology of data into account in subspace learning. Different from MLRSSC method, both BLLRR and MmCLRR methods are all committed to obtaining the global and local structures of manifold in multi-omics data by introducing manifold regularization constraint into LRR. Therefore, next, we compare MLRSSC with BLLRR and MmCLRR. For the convenience of comparison, as shown in Fig. 3, the histograms of clustering results on these three methods are given. From Fig. 3, it can be find that the values of all measures on method BLLRR and MmCLRR are higher than those on method MLRSSC. This indicates that the local geometry structure embedded in high-dimensional data is very vital to subspace segment problem. Preserving the local structure information of high-dimensional data during spatial mapping is helpful to smooth the manifold structure of the data in low-dimensional space and improve the subspace learning performance of the low-rank representation algorithm.

Thirdly, we compare MmCLRR with BLLRR. As be seen in Fig. 3, the experiment results of MmCLRR are better than BLLRR, especially on HNSC data set. First, for multi-omics analysis, the frameworks of the two methods are different. BLLRR is a method based on integrated multi-omics data. In BLLRR, the multi-omics data are integrated to form a comprehensive data matrix across omics. And the consistent low-dimensional subspace representation shared by multi-omics data is learned from the integrated data by imposing different penalty constraints on different omics data. MmCLRR is a method of multi-views learning. In MmCLRR, it is considered that the subspace representation from different views should be consistent. MmCLRR jointly learns the representation matrix of each view by enforcing the balance constraint between different views. In addition, if BLLRR is employed to single omics data, the objective of MmCLRR is transformed into $\min \left\| {\mathbf{Z}} \right\|_{*} + \gamma _{1} \left\| {{\mathbf{Z}}^{{}} } \right\|_{1} + \gamma _{2} \left\| {\mathbf{E}} \right\|_{{2,1}} + \gamma _{3} tr\left( {{\mathbf{ZL}}^{{}} {\mathbf{Z}}^{T} } \right){\text{ }}s.t.{\text{ }}{\mathbf{X}} = {\mathbf{XZ}} + {\mathbf{E}}$, that is, MmCLRR method is changed into BLLRR method. Similarly, when analyzing single omics data, the objective of MmCLRR method will become $\min \left\| {\mathbf{Z}} \right\|_{*} + \gamma _{1} \left\| {{\mathbf{Z}}^{{}} } \right\|_{1} + \gamma _{2} \left\| {\mathbf{E}} \right\|_{{2,1}} + \gamma _{3} tr\left( {{\mathbf{ZL}}^{{}} {\mathbf{Z}}^{T} } \right)$ $s.t.{\text{ }}{\mathbf{X}} = {\mathbf{XWZ}} + {\mathbf{E}},{\mathbf{WW}}^{T} = {\mathbf{I}}$. Obviously, the only difference between the two methods for single view is that the dictionary is constructed differently. BLLRR uses the original data as dictionary, which is fixed in iterative learning. And, MmCLRR applies the idea of concept factorization to construct dictionary matrix, which is constantly updated in learning. According to the above analysis, the clustering advantage of MmCLRR can be attributed to two points. One is that the multi-views learning model is more suitable for cross group analysis than the analysis model on integrated data. Another point is the successful modeling of dictionary by concept idea.

Finally, the MmCLRR approach is compared with MLRSSC. These two methods are basically consistent in the framework and main ideas for multi-view processing. There are two differences between them. On the one hand, compared with MLRSSC, manifold constraint is introduced into MmCLRR. On the other hand, the construction methods of dictionary are different. As mentioned above, MmCLRR takes the linear combination of original data as dictionary to update the dictionary matrix with the algorithm optimization, while MLRSSC uses original data as the fixed dictionary. From Fig. 3, we can see that the clustering advantage of MmCLRR method is much larger than that of BLLRR method. This fully shows that both manifold constraint and dictionary modeling make the low-rank representation matrix obtained by MmCLRR better distinguishable in subspace separation.

### The setting of parameters

In MmCLRR method, there are four regularization parameters $\gamma _{1}$, $\gamma _{2}$, $\gamma _{3}$ and $\gamma ^{{\left( v \right)}} (v = 1,2,3)$. As mentioned in the previous section, there is no prior knowledge to prove which omics data are more important in low dimensional learning. So we think that the proportion of each omics data in MmCLRR model is the same, and we use the same adjustment parameter $\gamma$ for all the three omics data, i.e., $\gamma {\text{ = }}\gamma ^{{\left( 1 \right)}} {\text{ = }}\gamma ^{{\left( 2 \right)}} {\text{ = }}\gamma ^{{\left( 3 \right)}}$. In our experiment, the parameters are set by grid search, and the parameter values are shown in Table 4.

**Table 4:** The paremeter values of MmCLRR on each experimental data set

Multi-omics data	$\gamma _{1}$	$\gamma _{2}$	$\gamma _{3}$	$\gamma$
HNSC	10^–1^	10^4^	10^–1^	10^0^
COAD	10^–1^	10^–1^	10^–1^	10^2^
ESCA	10^0^	10^–1^	10^2^	10^2^

## Discussion

MmCLRR is a novel multi-view integration analysis framework based low-rank decomposition. Our main contribution is to model dictionary matrix by concept factorization, which enables the dictionary matrix to update with subspace learning, thus enhancing the ability of dictionary to describe subspace. The comparative experiment of MmCLRR with other four multi-view methods is given on real multi-omics data. And the experiment results indicate that MmCLRR has a good performance in subspace clustering. In our experiment MmCLRR treats all omics data equally, so the parameter $\gamma ^{{\left( v \right)}}$, balancing the consistency of low-rank representation of different views, is set to the same. If different views are of different importance in the analysis, the parameter $\gamma ^{{\left( v \right)}}$ should be set to different values, which may increase the difficulty of parameter adjustment. Therefore, the increasing number of multi-view and the difference of their importance will be the main challenges for MmCLRR method.

## Conclusions

In this study, we develop a multi-view low-rank subspace clustering method, named as MmCLRR, to analyze caner multi-omics data. MmCLRR aims to achieve the consistent low-rank representation from multi-view data by balancing the consistency of different views. In our method, concept factorization is adopted to model dictionary. That is, the dictionary is constructed as the combination of the original data. Furthermore, the manifold regularization is introduced into our method to grasp the local structural relationship within the data. So, MmCLRR can capture the global and local structure of submanifold shared by multi-view data more efficiently. Finally, we adopt the proposed method to cluster cancer samples based on multi-omics data from TCGA. The experimental results demonstrated that our method can outperform the state-of-the-art multi-view approaches. In the future, we will promote the application of MmCLRR in other fields of cancer research.

## Data Availability

The datasets used in this study can be found in the [The Cancer Genome Atlas (TCGA)] https://cancergenome.nih.gov/.

## Abbreviations

LRR: Low-rank representation
MmCLRR: Multi-view manifold regularized compact low-rank representation
LADMAP: Linearized alternating direction method with adaptive penalty
